# 1-L Transcription in Alzheimer’s Disease

**DOI:** 10.3390/cimb44080243

**Published:** 2022-08-09

**Authors:** Jozef Nahalka

**Affiliations:** 1 Institute of Chemistry, Centre for Glycomics, Slovak Academy of Sciences, Dubravska Cesta 9, SK-84538 Bratislava, Slovakia; nahalka@savba.sk; 2 Institute of Chemistry, Centre of Excellence for White-Green Biotechnology, Slovak Academy of Sciences, Trieda Andreja Hlinku 2, SK-94976 Nitra, Slovakia

**Keywords:** Alzheimer’s disease, β-amyloid peptide, protein–RNA recognition code, bioinformatics method, identified genes

## Abstract

Alzheimer’s disease is a very complex disease and better explanations and models are needed to understand how neurons are affected and microglia are activated. A new model of Alzheimer’s disease is presented here, the β-amyloid peptide is considered an important RNA recognition/binding peptide. 1-L transcription revealed compatible sequences with AAUAAA (PAS signal) and UUUC (class III ARE rich in U) in the Aβ peptide, supporting the peptide–RNA regulatory model. When a hypothetical model of fibril selection with the prionic character of amyloid assemblies is added to the peptide-RNA regulatory model, the downregulation of the PI3K-Akt pathway and the upregulation of the PLC-IP3 pathway are well explained. The model explains why neurons are less protected from inflammation and why microglia are activated; why mitochondria are destabilized; why the autophagic flux is destabilized; and why the post-transcriptional attenuation of the axonal signal “noise” is interrupted. For example, the model suggests that Aβ peptide may post-transcriptionally control ELAVL2 (ELAV-like RNA binding protein 2) and DCP2 (decapping mRNA protein 2), which are known to regulate RNA processing, transport, and stability.

## 1. Introduction

**Figure 1:**
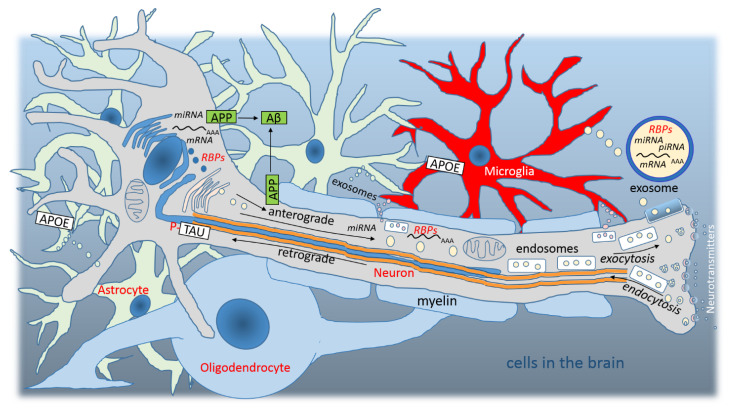
Graphic representation of cells in the brain. Nerve mRNAs and RBPs are transferred from the nucleus via the axon (exosomal pathway, anterograde), and glial mRNAs and RBPs are transferred from the extracellular space to the nucleus (endosomal pathway, retrograde). Dysregulation of APP/Aβ, TAU, and APOE functions are the basic three characteristics of AD. Due to the dysregulation of these three genes/proteins, disruption of axonal transport can be observed in AD.

Alzheimer’s disease (AD) was discovered in 1906 and today is classified as the most common form of age-related dementia [1,2]. Neuritic plaques composed of extraneuronal aggregations of β-amyloid peptide (Aβ), neurofibrillary tangles consisting of intraneuronal aggregations of hyperphosphorylated microtubule-associated TAU protein, and APOE gene mutations have been identified as three basic hallmarks of AD [2,3]. However, the pathological mechanisms of AD progression are a slightly more complex process [3]. Extraneuronal deposition of Aβ aggregates and intraneuronal deposition of TAU correspond well with neurodegeneration and progressive cognitive impairment in autosomal dominant AD, but are less consistent with sporadic AD [4]. In AD, almost all cell types (Figure 1) of the human brain are affected. However, the contribution of each cell type to the pathogenesis of AD is likely to be influenced by genetic risk factors and unknown environmental variables [5].

Recent characterization of the genetic environment of AD has confirmed the involvement of β-amyloid-TAU pathways, and highlighted the implication of microglia [6]. Microglia are important immune cells of the central nervous system (CNS) [7], which together with other glial cells communicate with neurons mainly through exosomes (Figure 1) [8]. The cellular mechanism involved in exosome biogenesis is associated with metabolism and transport in membrane organelles including the endoplasmic reticulum, trans-Golgi network, endosomes, lysosomes, and autophagosomes [8]. Exosomes are small extracellular vesicles that carry host cell components, particularly messenger RNA (mRNA), micro RNA (miRNA), piwi-interacting RNA (piRNA), non-coding RNA, and RNA binding proteins (RBPs) [8,9]. In fact, RNA is transported throughout the axon, and membrane vesicles control transport, control the sorting of mRNA populations, and even participate in mRNA translation (Figure 1) [9]. Through endosomes and multivesicular bodies, the pathway of anterograde exocytosis is linked to the pathway of retrograde endocytosis, so that “signaling endosomes” generated on the plasma membrane can control intracellular processes as well as exocytosis, especially mRNA transport and translation (Figure 1) [9]. Late endosomal mRNA transport has been shown to be associated with several mRNAs involved in promoting mitochondrial function, and conversely, mitochondria have been shown to be another platform for regulating the translation of localized mRNA molecules [9]. Dysregulation of these processes can lead to axonal degeneration, and the observed overcrowding of axonal transport appears to be a central point of neural network disintegration in AD [10].

**Figure 2:**
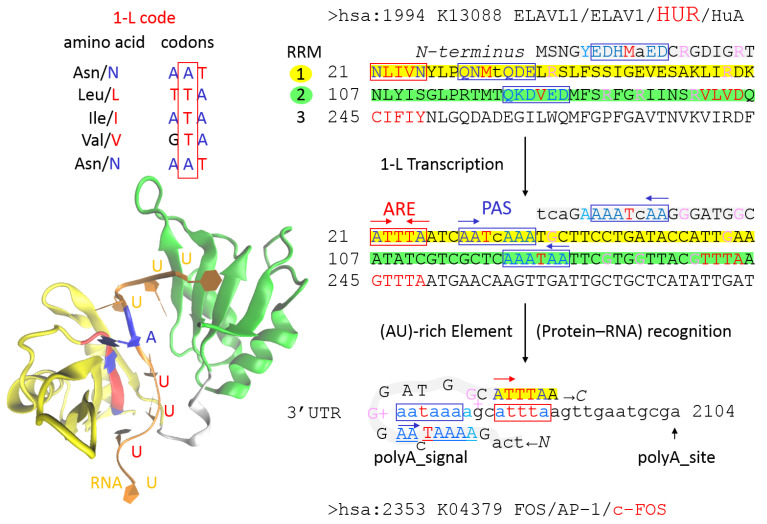
1-L code and 1-L RBP HUR transcription. HUR is a key regulator of cellular mRNAs containing adenylate/uridylate-rich (ARE) elements, for example, it regulates the stability of FOS mRNA. It has three RNA recognition motifs (RRM), the first is the most conserved and can be accurately transcribed into ARE and PAS sequences by 1-L transcription. The structure of RRM1-RRM2 (4ed5) shows that the sequence 21NLIVN is involved in ARE recognition, but the uncrystallized disordered N-terminus may also be involved in recognition, based on the obtained 1-L compatibility between the HUR N-terminus and the c-FOS 3′UTR.

1-L transcription is a simple computational method successfully tested on the life cycle and pathogenesis of SARS-CoV-2 [11,12]. In this method, the amino acid sequence of the RNA binding protein (RBP) was transcribed with a 1-L protein-RNA recognition code into compatible RNA sequences, which were then used for classical BLASTn screening in the human transcriptome to identify genes that can be post-transcriptionally regulated by the analyzed RBP. It has previously been shown that toxic mRNAs involved in neurodegeneration usually contain repeating nucleotide sequences that sequester RBPs specializing in these repeating sequence motifs, and sequestered RBPs can be identified by transcribing RNA-repeating motifs into amino acid sequences by using 1-L and 2-L protein-RNA recognition codes [13]. In the cell, RBPs have the function of “smoothing the transcriptional signal”, regulating all aspects of RNA life, and one RBP can recognize hundreds of transcripts and create extensive regulatory networks [14]. Not surprisingly, RBPs are more evolutionarily conserved than transcription factors [14]. Classical RBPs are characterized by the presence of one or more ordered RNA binding domains (RBDs), but other RBPs often lack recognizable RBDs and contain intrinsically disordered regions that are directly involved in RNA binding [14]. For example, the RBPs WDR33 and CPSF30 each use intrinsically disordered regions to read the PAS signal, a hexameric AAUAAA poly(A) signal that defines the pre-mRNA processing site at the 3′ end, cleavage, and polyadenylation [12]. WDR33 has been shown to have a highly conserved N-terminal sequence that is disordered and can be transcribed by the 1-L code into the PAS signal in reverse mode. For example, the *S. cerevisiae* WDR33 homologue has the sequence 12QNQIQQ, which can be transcribed by the 1-L code into the AAUAAA sequence in reverse mode [12]. The 1-L protein-RNA recognition code means that RBPs use at least one amino acid sequence that is exactly compatible with the recognized RNA nucleotide sequence, according to the key one amino acid per nucleotide (1-L),defined by the type of nucleotide at the second position in the amino acid codon [12,15,16]. As another example, Figure 2 shows the transcription of ELAVL1/HUR RBP by code 1-L, one of the best characterized RBPs. The human R antigen (HUR) binds more than 26,000 RNA binding sites, which are found mainly in 3′ untranslated regions (3′UTRs), but many of which are intronic [17]. HUR is a key regulator of cellular mRNAs containing adenylate/uridylate-rich elements (ARE) [18]. There are three classes of AREs: Class I contains several AUUUA motifs scattered along the 3′UTR; Class II contains overlapping copies of the AUUUA; Class III is rich in U [18]. HUR has three RNA recognition (RRM) motifs involved in ARE binding (Figure 2), the first of which is the most evolutionarily conserved, from *S. cerevisiae* to *Homo sapiens* (Figure S1). By applying 1-L transcription, the 21NLIVNYLPQNMtQDE amino acid sequence of RRM1 was transcribed into ATTTAATCAATcAAA, where ATTTA represents the ARE and AATcAAA PAS sequence spaced with c/t (Figure 2). The crystal structure shown in the figure shows that the 21NLIVN sequence is involved in ARE binding, but in disordered forms the N-terminus of HUR outside of RRM1 is likely to promote binding, as shown for FOS/AP-1/c-FOS mRNA (Figure 2). Essentially two arginine residues are involved in the disordered RNA binding mode, and the ARE and PAS amino acid recognition sequences participate in nucleotide sequence reading (Figure 2). Interestingly, the neural homologue HUB/ELAVL2 was able to initiate cytosolic translocation of HUR from the nucleus to the cytosol and simultaneously regulate the expression of basal c-FOS mRNA [19]. In addition, HUD/ELAVL4 interacted with the 3′UTR of mRNA encoding amyloid precursor protein (APP) and increased the half-life of this mRNA [20].

**Figure 3:**
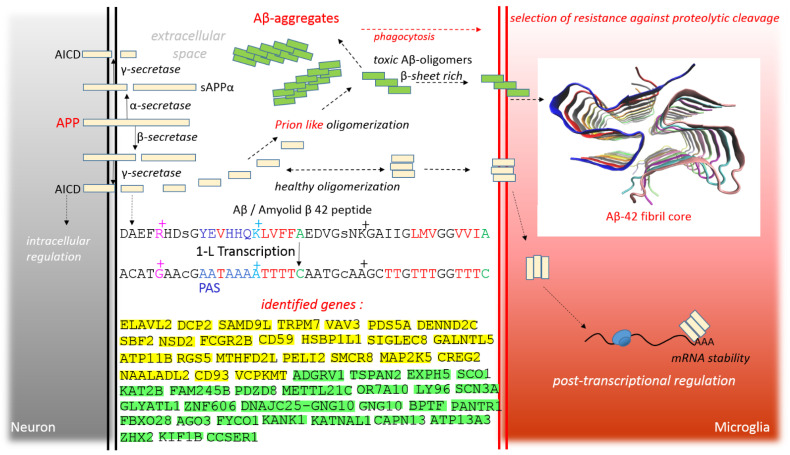
Two different pathways of APP processing in PM, and healthy oligomerization of Aβ peptide to elicit immune responses versus toxic prion oligomerization. The 1-L transcription of the Aβ peptide identifies the PAS sequence and the TTTC sequence (U-rich ARE class III). Genes identified by 1-L transcription, green highlights show alignments with complement sequence (post-transcriptionally repressed), yellow highlights show alignments with reverse complement sequence (post-transcriptionally promoted).

APP is a widely expressed and distributed type I transmembrane protein that has been primarily identified as a source of extracellular senile plaques in the AD brain [3,21]. In PM, there are two different pathways of APP processing: non-amyloidogenic and amyloidogenic [21]. In non-amyloidogenic processing, APP is cleaved by α-secretase to the soluble amino-terminal ectodomain of APP (sAPPα) and the C-terminal fragment of C83. Subsequent cleavage of C83 by the γ-secretase complex forms the intracellular domain of APP (AICD) and a short fragment called p3 (Figure 3). In amyloidogenic processing, APP is cleaved by β-secretase to sAPPβ and the C-terminal fragment of C99. Subsequent cleavage of C99 by the γ-secretase complex produces AICD and Aβ peptide (Figure 3) [21]. In general, non-amyloidogenic processing is upregulated in autism [22] and amyloidogenic processing is upregulated in AD (amyloid cascade hypothesis) [3]. In other words, sAPPα signaling may be important for brain growth and Aβ peptide signaling may be important for immune responses in the brain. In fact, the Aβ-42 peptide has convincing structural similarities to antimicrobial peptides, as well as sequence similarities to a specific family of bacterial bacteriocins [23]. In light of this, Aβ peptides have been proposed as elicitors of the immune response against microbes [23], and even Aβ-42 peptide has been shown to protect against microbial infection in mouse and worm models of AD [24].

Amyloid fibrils are formed in the AD brain, especially during Aβ-42 aggregation [25]. In in vitro experiments, several μM of Aβ is usually required for aggregation and fibril formation, although the physiological concentration of Aβ is less than nM. In light of this, a hypothetical model of fibril selection was proposed. An internal feature of the model is the prionic nature of amyloid clusters and, of course, the aspect of transmission from one organism to another is a property of prions [25]. It appears that two pathways can be interpreted for oligomerization of the Aβ peptide, healthy oligomerization to elicit immune responses, or prion-like toxic oligomerization (Figure 3).

Regarding the induction of immune responses by microglia, this study investigated the Aβ peptide as a potential small RBP. By applying the 1-L transcription of the Aβ peptide, it was possible to identify the PAS sequence and the TTTC sequence (U-rich ARE class III) in Aβ (Figure 3). Healthy oligomerization of the Aβ peptide could be required for the cooperative assembly of Aβ oligomers for RNA binding (Figure 3). As will be shown later, the spectrum of genes identified gives credibility to the methodological approachemployed to identify genes and to present the model that the Aβ peptide signal may affect protein–RNA homeostasis in microglia and neurons.

## 2. Method

The motivation for the concept is explained in the introduction, above. The 1-L protein–RNA recognition code means that RBPs use at least one amino acid sequence that is exactly compatible with the recognized RNA nucleotide sequence, according to the one-letter key, one amino acid per nucleotide (1-L), and the nucleotide is defined by the type of nucleotide in the second position in amino acid codon (Figure 2). In this method, a peptide or protein sequence was simply transcribed into the second codon nucleotide of each amino acid of the protein. The resulting imaginary RNA sequence was then used for a BLASTn search in the human transcriptome, to identify genes that can be post-transcriptionally regulated by the protein of interest. The procedure is very simple, but S (Ser) has two transcription options, transcription to C (cytidine) or G (guanosine), so that two nucleotide sequences were obtained for each amino acid sequence, one with S-C transcription and the other with S-G transcription. Another point is that 5′-RNA reading can be performed with the N-(AA)n-C amino acid sequence or the reverse C-(AA)n-N amino acid sequence (Figure 2), so that transcription was performed for two amino acid sequences, one for N-(AA)n-C and the other for C-(AA)n-N. In summary, four nucleotide sequences were obtained (please see Supplementary Sequence Information).

BLASTn screening in the human transcriptome was performed as a standard nucleotide blast in NCBI (https://blast.ncbi.nlm.nih.gov/Blast.cgi; accessed on 8 August 2022) separately for four nucleotide sequences. “Genomic + transcript databases” and “human genomic plus transcript”, “slightly similar sequences” (blast algorithm), word size 7, maximum number of target sequences 500, and expected threshold 100 were used for the search. However, the user may choose word size 7 or higher, or change another parameter to take into account the level of reliability: Perfect pairing—low number of hits; imperfect pairing—high number of random hits and false positives.

Alignments with the gene transcript sequence were considered repressive (green in the figures) and alignments with the reverse complement sequences were considered promotive (yellow in the figures).

## 3. Results

The rationale and summary of the identified genes are shown in Figure 3. In this study, comparison with the complement sequence was considered responsible for post-transcriptional repression, because if the regulatory RBP/peptide interferes with mRNA, it suppresses translation and possibly initiates degradation. Conversely, alignments with reverse complement sequences were considered responsible for post-transcriptional promotion, because regulatory small RNAs or microRNAs with reverse complement sequences pair and bind to mRNA to allow post-transcriptional repression, then supporting translation if the regulatory RBP/peptide interferes with regulatory RNA. In fact, it may be the other way around. RBPs control the whole of RNA life: splicing, capping, polyadenylation, transport, localization, translation, degradation, and the result of RBP–RNA interactions may be different for different RNAs, different stages of RNA life, and different sites in one RNA molecule. For example, FMRP RBP acts as a repressor of APP mRNA, but hnRNPC RBP is able to stabilize it, thereby facilitating APP expression [26].

Nevertheless, as mentioned above, alignments with the gene transcript sequence were considered repressive (green in the figures) and alignments with complement reverse sequences were considered supportive (yellow in the figures). According to the hypothesis, aggregation and sequestration of microRNAs into RNP granules promotes translation, and aggregation and sequestration of mRNA into RNP granules suppresses translation.

### 3.1. Identified Genes/Proteins with Functions in the Plasmatic Membrane and PM-Cytosol Interface

**Figure 4:**
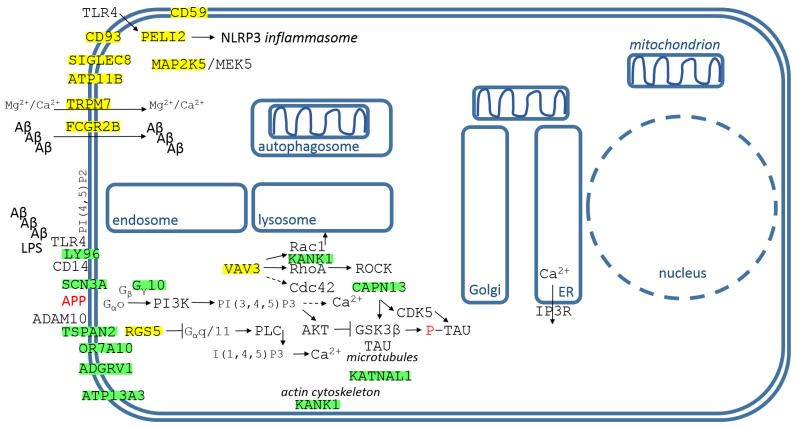
Identified genes/proteins with functions in the plasma membrane and at the PM-cytosol interface. The description of genes/proteins is given in the text from the top left corner down. Green highlights show alignments with the complement sequence (post-transcriptionally repressed), yellow highlights show alignments with the reverse complement sequence (post-transcriptionally promoted).

The genes/proteins are displayed in Figure 4, with their descriptions from top left corner down.

CD59 (complement defense 59) is a cell-surface glycophosphoinositol (GPI)-anchored protein that prevents complement membrane attack complex (MAC) assembly. CD59 protein levels are significantly decreased in the frontal cortex and hippocampus of AD compared with nondemented elderly patients [27].

CD93 is an important neuro-immune regulator to control central nervous system inflammation; CD93^−/−^ mice presented a more robust brain and spinal cord inflammation characterized by increased numbers of infiltrating microglia [28].

PELI2 is the E3 ubiquitin ligase that mediates priming of the NLRP3 inflammasome [29]. It was identified as a potential driver gene in AD by transcriptomics data [30].

SIGLEC8 (sialic acid binding Ig-like lectin 8) is a CD33-related SIGLEC [31]. A monoclonal antibody with SIGLEC CD33 antagonistic activity is currently being evaluated in patients with AD [31]. Mice do not express Siglec-8, but Siglec-F is a likely functional orthologue of Siglec-8. Siglec-F was upregulated on a subset of reactive microglia in models of neurodegeneration, and it was observed that Siglec-F is dependent on Aβ deposition at early AD stages [32,33].

MAP2K5/MEK5 is an element of the MAPK-family intracellular signaling pathways, which respond to CNS modulators such as the brain-derived neurotrophic factor (BDNF) and nerve growth factor (NGF) [34]. Interestingly, hyperglycemia regulates microglia polarization into an increasingly proinflammatory subtype, which can be suppressed by sustained activation of ERK5 by transfected MAP2K5/MEK5 [35].

ATP11B is P4-ATPase membrane protein that serves as lipid flippase, regulates membrane asymmetry, and modulates the morphology of neural stem cells. ATP11B deficiency leads to impairment of hippocampal synaptic plasticity [36].

TRPM7 (transient receptor potential cation channel) is a chanzyme composed of an ion channel with an α-kinase domain on its C-terminus [37]. Mg^2+^ deficiency is linked to AD, and extracellular Mg^2+^ enters endothelium mainly through the TRPM7 channel (plus MagT1 transporter). Mg^2+^ also regulates endothelial barrier functions through TRPM7 [38]. It was proposed that tetraspanin CD82-TRPM7-Numb signaling mediates age-related cognitive impairment, and CD82 overexpression promoted Aβ peptide secretion [39].

FCGR2B (Fc gamma receptor IIb) mediates Aβ neurotoxicity and memory impairment in AD [40]. FCGR2B binds to the Aβ peptide, mediates the endocytosis, and results in TAU hyperphosphorylation through the deregulation of phosphoinositide signaling. FCGR2B2 variant was observed to be critical in the neuronal uptake of pathogenic Aβ for neurotoxicity in vitro and in AD model mice [41].

LY96/MD-2 (lymphocyte antigen 96) is a secreted large polymeric protein that efficiently confers lipopolysaccharide sensitivity to Toll-like receptor 4 (TLR4) [42]. Tri-molecular receptor complex consisting of TLR4, LY96/MD-2 and CD14 is necessary for full cellular activation by aggregated Aβ (Figure 4) [43].

VAV3 (vav guanine nucleotide exchange factor 3) belongs to the GEFs (guanine nucleotide exchange factors) that catalyze the exchange of guanosine diphosphate (GDP) by guanosine triphosphate (GTP) on their target proteins, such as Ras homolog family member A (RhoA), Ras-related C3 botulinum toxin substrate 1(Rac1), and cell division control protein 42 homolog (Cdc42), (Figure 4). Interestingly, VAV3-deficient astrocytes enhanced the dendritic development of hippocampal neurons [44].

SCN3A (sodium voltage-gated channel alpha subunit 3) is robustly PM-channel expressed across human cortical regions during fetal periods, but is downregulated after birth and undetectable latter. However, it is upregulated in response to various insults to the nervous system, including nerve injury, and individuals with pathogenic SCN3A variants display aberrant cerebral cortical development and speech deficits [45].

GNG10 (G protein subunit gamma 10) is involved in G protein-coupled receptor signaling. In GPCR signalling, ligand-bound GPCRs activate heterotrimeric G proteins, inducing the exchange of GDP for GTP, the formation of a GTP-bound Gα subunit, and the release of a Gβγ dimer. The G protein subunits then activate specific secondary effector molecules, especially the PI3K/Akt pathway [46]. Interestingly, it seems that sAPPα-APP interactions induce the PI3K/Akt pathway via Gαo activation, and that APP works like GPCR and sAPPα works like its agonist (Figure 4) [47]. GNG10 is differentially expressed in the hippocampus of AD [48,49].

TSPAN2 (tetraspanin 2) is one of the less characterized members of the tetraspanin superfamily, and may contribute to the early stages of oligodendrocyte differentiation into myelin-forming glia [50]. Interestingly, tetraspanin 2 interacts with α-secretase ADAM10 [50]. Transmigration of tetraspanin 2 siRNA via microglia-derived exosomes across the blood brain barrier model modified the production of immune mediators by microglia cells: it decreased chemokine CXCL12, chemokine receptor CXCR4, and interleukins IL-13 and IL-10, and increased Fc gamma receptor 2A (FCGR2A) [51].

RGS5 (regulator of G protein signaling 5) terminates G-protein-coupled signaling cascades which control contractile responses of vascular smooth muscle cells (VSMC), shifts GPCR signaling from Gα_q/11_-mediated calcium-dependent contraction towards Gα_12/13_-mediated RhoA signaling and VSMC activation, and promotes arterial growth (Figure 4) [52]. Cerebral atherosclerosis is related to AD.

OR7A10 (olfactory receptor family 7 subfamily A member 10) is GPCR expressed by olfactory sensory neurons located in the olfactory epithelium in the nasal cavity. Olfactory dysfunction is known in AD [53]. One study identified OR7A10 among genes associated with cortical thickness in AD [54].

ADGRV1 (adhesion G protein-coupled receptor V1) is the largest aGPCR in terms of total amino acids. It is highly expressed in the stereocilia of the cochlea and its mutations cause Usher syndrome type 2C (deafness and blindness) [55]. ADGRV1 was chosen as the cell type marker for astrocytes in AD cell type-specific transcriptomic studies [56,57]. It seems that ADGRV1 works like a mechanosensor at focal adhesions, regulating cell spreading and migration [58].

ATP13A3 (ATPase 13A3) is a major component of the enigmatic mammalian polyamine transport system. Cellular polyamine homeostasis must be tightly controlled; excessive concentrations of polyamines induce cellular toxicity [59]. ATP13A3 was significantly upregulated in transcriptomic profiling of myeloid cells in AD brains compared to controls [60].

KANK1 (KN motif and ankyrin repeat domains 1) was first described in renal cell carcinomas where it was identified as a tumor suppressor. It negatively regulates actin polymerization and cell migration through Rho GTPases RhoA and Rac1. KANK1 phosphorylated by AKT recruits 14-3-3 protein and inhibits RhoA activation, it also interacts with insulin receptor substrate 53 kDa (IRSp53) and inhibits Rac1 signalling [61].

CAPN13 (calpain 13) belongs to the group of specific Ca^2+^-dependent cysteine proteases involved in the neuropathogenesis of AD. Calpains cleave the microtubule-associated protein TAU, proteolytic fragments of TAU detached from microtubules have an increased propensity to phosphorylate and aggregate into neurofibrillary tangles [62]. Calpain 1 also truncates and activates GSK3β, which leads to phosphorylation of TAU [63]. Calpain mediates the cleavage of p35 to p25 (the most potent activator of CDK5), and CDK5 activates GSK3β, which initiates phosphorylation of TAU (Figure 4) [64]. However, the exact biochemical properties and biological functions of CAPN13 are not known; it is localized into the mitochondria in addition to the cytosol [65].

KATNAL1 (katanin catalytic subunit A1 like 1) is one of the two major catalytic subunits of the microtubule-severing enzyme Katanin, together with KATNAL2. KATNAL1 is involved in neural morphology, and its knockdown enhances axon elongation [66]. TAU provides protection of microtubules to Katanin, and KATNAL1 is upregulated in TAU’s absence [66].

### 3.2. Identified Genes/Proteins with their Functions in the Membrane Organelles, Mitochondria, and Nucleus

**Figure 5:**
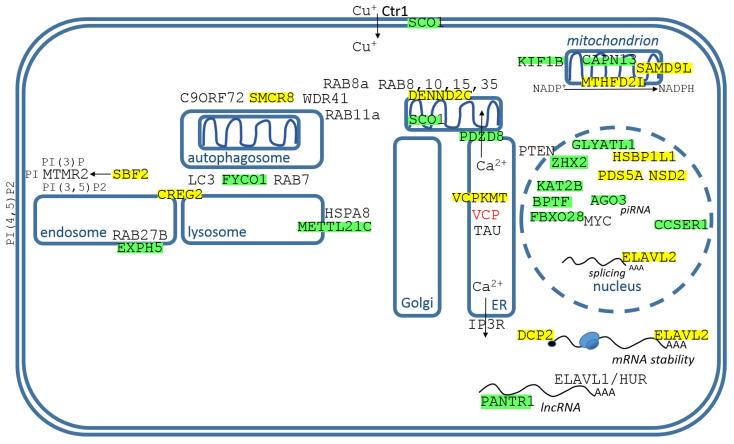
Identified genes/proteins with functions in membrane organelles, mitochondria, and nucleus. The descriptions of genes/proteins are given in the text, from endosomes to nucleus, from left to right. Green highlights show alignments with the complement sequence (post-transcriptionally repressed), yellow highlights show alignments with the reverse complement sequence (post-transcriptionally promoted).

The genes/proteins are displayed in Figure 5 and their descriptions are from endosomes to nucleus, left to right.

SBF2/MTMR13 (SET binding factor 2) belongs to the group of myotubularin-related proteins involved in regulating endolysosomal trafficking, namely MTMR2, MTMR13/SBF2, and MTMR5/SBF1. SBF2 mutations cause Charcot-Marie-Tooth disease type 4B2 (CMT4B2), a sensorimotor neuropathy; CMTs share similar features including a demyelinating neuropathy associated with reduced nerve conduction velocity and focally folded myelin [67]. MTMR2 acts as a phosphoinositide D3-phosphatase with phosphatidylinositol (PtdIns) 3-phosphate and PtdIns 3,5-bisphosphate as substrates, PI(3)P and PI(3,5)P2. SBF2/MTMR13 forms a tetrameric complex with MTMR2, resulting in a strong increase of the enzymatic activity of complexed MTMR2 [67].

EXPH5 (exophilin 5) is positive effector of RAB27B; knockdown of RAB27 or EXPH5 could inhibit the release of exosomes from HeLa cells [68]. In the transcriptomic profile of a human brain associated with aging, EXPH5 is downregulated among neurologically healthy individuals [69].

CREG2 (cellular repressor of E1A stimulated genes 2) is only detected in the brain, CREG1 mRNA is ubiquitously expressed, but until now only CREG1 has been characterized [70]. CREG1 is mainly localized in the endosomal–lysosomal compartment and has roles in macropinocytosis and clathrin-dependent endocytosis. Functionally, overexpression of CREG1 enhances macroautophagy/autophagy and lysosome-mediated degradation, whereas knockdown or knockout of CREG1 has opposite effects [70].

FYCO1 (FYVE and coiled-coil domain autophagy adaptor 1) is a RAB7 effector that binds to LC3 and PI3P to mediate microtubule plus end-directed vesicle transport [71]. LC3B is critical for the retrograde transport of autophagosomes within cells, its STK4-mediated phosphorylation regulates FYCO1 binding and the directional transport of autophagosomes (Figure 5) [72]. In the hallmark neuritic dystrophy of AD, autophagic vacuoles containing incompletely digested proteins selectively accumulate in focal axonal swellings, reflecting defects in axonal transport as well as autophagy [73].

SMCR8 (guanine nucleotide exchange protein) makes a stable complex with C9ORF72 and WDR41, which works as a Rab GEF for RAB8a and RAB11a, and regulates autophagic flux [74].

METTL21C (methyltransferase-like 21C) trimethylates heat shock protein 70kDa HSPA8 (HSP73) at Lys-561 to enhance its stability. HSPA8 has functions in chaperone-mediated autophagy [75]. Chemical inhibitors of Hsp70 ATPase activity led to rapid proteasome-dependent TAU degradation and activators preserved TAU levels, in a cell-based model [76]. HSPA8 and HSPA1A knock-down increases TAU and α-synuclein protein levels [77].

DENND2C (DENN domain containing 2C) is guanine nucleotide exchange factor for RAB8A, RAB8B, RAB10, RAB15, and RAB35 [78]. The DENN domain-bearing proteins comprise the largest family of Rab GEFs [78]. In a study of microglial depletion and repopulation with new and unprimed microglia in aged mice, normally age-decreased gene Dennd2c was reversed after microglial repopulation [79].

SCO1 (synthesis of cytochrome C oxidase 1) is a Cu metallochaperone, important for the cytochrome c oxidase assembly [80]. It is located in the inner mitochondrial membrane, where it transports Cu ions to the CuA site on COX2 [80]. Its other important role is controlling the localization and abundance of Ctr1, copper transporter-1, a plasmatic membrane protein which transports Cu over the membrane into the cytosol [80]. It is thought that copper dysregulation prevails in AD, and interactions between Aβ peptides and copper may form neurotoxic Aβ oligomers [81].

PDZD8 (PDZ domain containing 8) is required for calcium ion (Ca^2+^) uptake by mitochondria after synaptically induced Ca^2+^-release from ER [82]. PDZD8 is responsible for ER–mitochondria tethering [82]. Depletion of pdzd8 rescued the locomotor defects characterizing AD in a fly model over-expressing Aβ-42 [83].

VCPKMT/METTL21D (valosin containing protein lysine methyltransferase) methylates single lysine residue in VCP, which is an ATP-dependent chaperone [84]. VCP is involved in preventing protein aggregation and mediating the degradation of aberrant proteins by proteasome and autophagy. VCP is known to co-localize with TAU, and alterations in VCP cause aberrant accumulation of TAU [85].

PANTR1 (POU3F3 adjacent non-coding transcript 1) is long non-coding RNA that flanks the POU-domain gene family member Pou3f3 (Brn1), a key transcription factor involved in brain cortical development, and Pantr1 shares a bidirectional promoter with Pou3f3 [86]. PANTR1 is strongly expressed in the hippocampus. Ablation of the Pantr1 locus resulted in significant up-regulation of the neuronal progenitor markers and significant down-regulation of mature neural cell markers [86]. HuR’s activity is involved in neurosphere formation and maintenance of stemness. RBP ELAVL1/HUR (Figure 2) was found to bind U-rich regions in LincBRN1a (mouse homolog) [87].

DCP2 (decapping mRNA protein 2) controls the stability of RNAs and is the major mRNA decapping enzyme, >1800 human DCP2 substrates have been identified [88]. DCP2 and its activator DCP1 are consistently co-localized in cytoplasmic RNA granules called processing bodies [89].

KIF1B (kinesin family member 1B) is a kinesin motor protein implicated in the axonal transport of mitochondria and synaptic vesicles. KIF1B-α abrogation decreases the mean velocity and density of mitochondria along the axon during anterograde movement [10]; it is essential for mRNA localization in oligodendrocytes and development of myelinated axons [90]. Expression of KIF5A, KIF1B, and KIF21B at gene and protein level is significantly increased in AD [91].

SAMD9L (sterile alpha motif domain-containing 9-like) is one of the genes influencing cerebellar microglia clearance activity and phenotype [92]. A novel SCA49 spinocerebellar ataxia subtype has been described, caused by SAMD9L mutation, which triggers mitochondrial alterations, pointing to a role of SAMD9L in neurological motor and sensory functions [93].

MTHFD2L (methylenetetrahydrofolate dehydrogenase (NADP+ dependent) 2-like) is enzyme with bifunctional dehydrogenase/cyclohydrolase activity in mitochondria. It utilizes NADP^+^ as a cofactor to transform serine-derived methylene-THF to formyl-THF, which results in the collateral production of mitochondrial NADPH. It is clear that decreased NADPH causes failure to supplement endogenous antioxidants such as GSH, which weakens ROS uptake, promotes oxidative stress, and promotes development of AD [94].

GLYATL1 (glycine-N-acyltransferase like 1) is known to be involved in the detoxification of endogenous and exogenous xenobiotic acyl-CoAs in mammals. Induction of HSPs is regulated by trans-acting heat shock factors (HSFs) and cis-acting heat shock element (HSE) present at the promoter region of each heat shock gene. GLYATL1 protein activates the HSE signaling pathway [95].

ZHX2 (zinc fingers and homeoboxes 2) is the transcription factor that promotes the transcription of phosphatase and tensin homolog (PTEN) and alleviates NASH (advanced liver diseases) [96]. GPCRs, PI3K, and Rho signaling pathways regulate the cascades of TAU and Aβ in AD (Figure 4). The regulation of PI3K is carried out by PTEN phosphatase, which directly inhibits PI3K.

KAT2B/PCAF (lysine acetyltransferase 2B) stimulates the gluconeogenic program through increasing histone H3 acetylation at Lys 9 (H3K9Ac) and furthers potentiation of CRTC2 occupancy at CREB binding sites. Administration of a small molecule KAT2B antagonist lowered circulating blood glucose concentrations in insulin resistance, suggesting that this enzyme may be a useful target for diabetes treatment [97]. One study found that mice lacking KAT2B/PCAF were resistant to the detrimental effects of directly injected Aβ peptides, however, the same KAT2B/PCAF mutant mice were found to show impaired memory function [98].

BPTF (bromodomain PHD finger transcription factor), also known as fetal Alzheimer antigen (FALZ), is expressed in several isoforms, a shorter N-terminal isoform FAC1 (fetal Alzheimer’s clone 1), and oncogenic BPTF fusion proteins. FAC1 was first identified from amyloid plaques of AD patients. The full-length BPTF gene serves as the largest subunit of NURF, the founding member of the ATP-dependent chromatin remodeling complexes [99].

FBXO28 (F-box protein 28) is the substrate recognition adapter of SCF (SKP1/CUL1/F-box) E3 ubiquitin ligase complex, responsible for ubiquitination and proteasomal degradation. In the case of MYC-dependent transcription, non-proteolytic ubiquitination by SCF^FBXO28^ transmits CDK activity to the MYC function, FBOX28 is phosphorylated and activated at Ser 344 by CDK1/2, and SCF^FBXO28^ then promotes ubiquitination and stimulates MYC activity [100]. Interestingly, microRNA-146a negatively correlates with Aβ peptide, and FBXO28 is among microRNA-146a differentially expressed genes [101].

AGO3 (argonaute RISC catalytic component 3) is one of four argonaute proteins (AGO1–4) expressed in humans. AGO2 has been the best described and was long thought to be the only argonaute protein member with mRNA slicing activity. Nevertheless, AGO3, like AGO2, has a fully functional PIWI domain that when loaded with certain miRNAs is similar to an RNAse H domain [102]. PIWI-interacting RNAs (piRNAs, 26–30 nt in length) are small non-coding transcripts that are highly conserved across species and regulate gene expression through pre- and post-transcriptional processes. piRNAs togeather with miRNAs are strongly implicated in AD and their expression signatures can distinguish AD patients from controls [103].

There are no reports about the exact biological functions of HSBP1L1 (heat shock factor binding protein 1 like 1). Presumably, HSBP1L1 might suppress heat shock factor transcription under stress.

PDS5A (PDS5 cohesin associated factor A) is a cell-cycle-related gene. PDS5A is a nuclear protein and plays a role in the establishment, maintenance and dissolution of sister chromatid cohesion. It may contribute to tumorigenesis by interacting with p63 and promoting cell cycle progression [104]. Tocotrienol-rich fraction (TRF) supplementation was able to reduce fibrillar Aβ deposition in the hippocampus of an AD mouse model, and PDS5A was among the top 20 upregulated genes after six months of TRF supplementation [105].

NSD2 (nuclear receptor binding SET domain protein 2) is the principal histone methyltransferase that dimethylates histone H3 at lysine 36 (H3K36me2), a mark associated with active gene transcription. NSD2-driven tamoxifen-resistant cells and tumors displayed heightened pentose phosphate pathway activity, elevated NADPH production, and reduced ROS level, without significantly altered glycolysis [106].

CCSER1 (coiled-coil serine rich protein 1) is one of the genes influencing the rate of cognitive decline in patients with AD [107]. It is also one of the risk genes for AD, according to the hypothesis of long gene vulnerability, which provides a simple link between aging and the genetic landscape of AD [108].

ELAVL2 (ELAV-like RNA binding protein 2) is an ELAV1/HUR (Figure 2) homolog and belongs to the neuronal-specific mammalian embryonic lethal, abnormal vision-like (ELAVL)2, 3 and 4 RBPs, which are an RBP family based on homology to ELAV protein in Drosophila and regulate the splicing pre-mRNAs [109] and the transport, stabilization, localization, and translation of mRNAs [110]. ELAVL2 interacts with hnRNP K to control neuronal differentiation, and regulates axonogenesis via post-transcriptional interaction with genes involved in neurodevelopment, transport, localization, and cytoskeleton, including GAP43 [110]. *Apis mellifera* (bees) have only one elav/Hu family gene elavl2, expression and alternative splicing of which is required for learning and memory [111]. Regulation of transcript expression by ELAVL2 has been shown to be critical for neuronal function and clinically relevant to autism [112], and ELAV1/HUR and ELAV2/HUB are critical for the occurrence of MMP-9 mRNA stabilization upon neuronal activation [113]. In neurons, ELAVL proteins utilize AUF-1 as a co-partner to induce specific alternative splicing of APP [114]. ELAVL2 is also expressed in testis and plays post-transcriptional roles in the promotion of spermatogonia proliferation and inhibition of apoptosis by activating ERK and AKT pathways [115].

## 4. Discussion

As mentioned in the introduction, post-transcriptional regulation (RBPs) is more evolutionarily conserved than transcriptional regulation (TFs) [9]. In addition, regulation by transcription factors works at larger concentration intervals and provides much slower responses compared with post-transcriptional regulation by RBPs and miRNAs, which is fast and works at tight concentration intervals. In other words, the “noise” signal from the DNA is additionally transcribed into a “smooth” signal. In neurons in particular, long axonal transport aids in the post-transcriptional regulation of “noise” signals. In AD, axonal transport and post-transcriptional regulation of “noise” are dysregulated and memory loss is observed.

This study investigated how the Aβ peptide could be involved in the post-transcriptional regulation of signal “noise”. 1-L transcription revealed sequences compatible with PAS and TTTC (class III ARE rich in U) in the Aβ peptide (Figure 3), supporting the peptide–RNA regulatory model. According to the presented model, Figure 4 shows dysregulated genes/proteins with functions in the plasma membrane and PM–cytosolic interface, and Figure 5 shows dysregulated genes/proteins with functions in membrane organs, mitochondria, and the nucleus. According to the presented model, the Aβ peptide gently promotes the genes/proteins obtained by alignment with the reverse complement sequence (yellow highlights), and gently represses the genes/proteins obtained by complement sequence alignment (green highlights).

In healthy individuals, the Aβ peptide gently promotes CD59 and CD93 and protects against inflammation of the nervous system, promotes hippocampal synaptic plasticity (ATP11B), gently promotes Mg^2+^ transport into the cell (TRPM7 channel), gently blocks TLR4 sensitivity in microglia by suppressing LY96, slightly supports the PI3K-Akt pathway and blocks the PLC-IP3 pathway, tightly controls homeostasis of cellular polyamine (ATP13A3), tightly controls the microtubule-cleaving Katanin (KATNAL1), and represses the specific Ca^2+^ dependent protease CAPN13 (Figure 4). Figure 5 shows that the Aβ peptide gently represses Ca^2+^ transport to mitochondria (PDZD8) and the mitochondrial activity of CAPN13, supports mitochondrial NADPH (MTHFD2L) and protects mitochondria from ROS, tightly controls the kinesin motor protein KIF1B involved in axonal transport of mitochondria and synaptic vesicles, tightly regulates autophagic flux (FYCO1, SMCR8, METTL21C), and tightly regulates Cu^+^ transport across the plasmaticmembrane to the cytosol and mitochondrial Cu^+^ transport into CuA site on COX2 (SCO1). In the nucleus, the Aβ peptide suppresses TF ZHX2, which promotes transcription of PTEN phosphatase, which directly inhibits the PI3K-Akt pathway. Aβ suppresses KAT2B/PCAF, which stimulates the gluconeogenic program; Aβ also promotes the major histone methyltransferase NSD2 (H3K36me2), associated with increased NADPH levels and decreased ROS levels; and Aβ post-transcriptionally supports RBP ELAV2/HUB, which is required for learning and memory in bees [111] (Figure 5).

**Figure 6:**
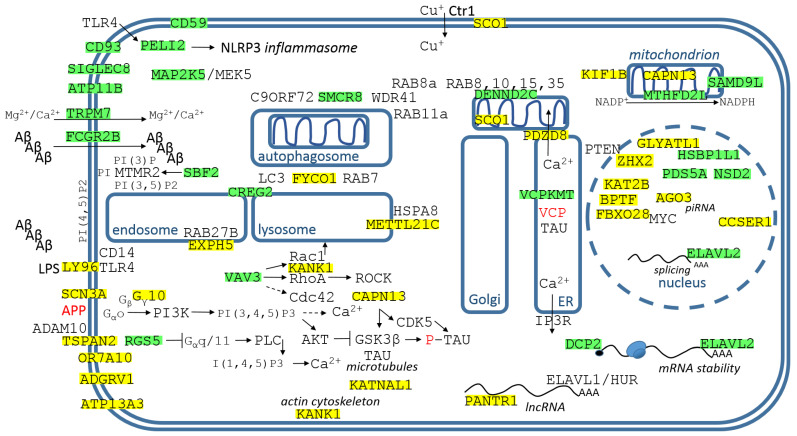
The situation when the prion-like oligomerization consumes the Aβ signal peptide. Green highlights show the alignments with the reverse complement sequence (post-transcriptionally not promoted), yellow highlights show the alignments with the complement sequence (post-transcriptionally not repressed).

As mentioned in the introduction, two pathways can be interpreted for oligomerization of the Aβ peptide, i.e., healthy oligomerization for post-transcriptional “signal noise suppression” and prion-like toxic oligomerization (Figure 3). In patients with AD, prion oligomerization sequesters the released Aβ peptides and blocks post-transcriptional regulation by “healthy” Aβ oligomers. In this case, alignments with the reverse complement sequence are not post-transcriptionally promoted, and alignments with the complement sequence are not post-transcriptionally repressed (Figure 6). Figure 6 actually represents a mirror image of Figure 4 and Figure 5, “green has become yellow and yellow has become green”.

In the presented AD model, TF ZHX2 and GNG10 are supported, PTEN is upregulated and G_α_o is complexed, and then the PI3K-Akt pathway is inhibited; RGS5 is suppressed, the PLC-IP3 pathway is supported and subsequently IP3 induces Ca^2+^ release from the ER. Activated calpains cleave TAU, proteolytic fragments of TAU separated from microtubules, having an increased tendency to be phosphorylated, are aggregated into neurofibrillary tangles [62], and downregulation of the PI3K-Akt pathway activates GSK3β, leading to phosphorylation of TAU [63]. APP appears to function as a GPCR and sAPPα acts as its agonist [47], but in this case the signaling is redirected to the PLC-IP3 pathway (Figure 6). In this AD model, the Aβ peptide does not support CD59 and CD93, and does not protect neurons from inflammation; it does not support hippocampal synaptic plasticity (ATP11B); it does not support transport of Mg^2+^ to neurons (TRPM7 channel); and it does not control neuronal polyamine homeostasis (ATP13A3). However, LY96/MD-2 is promoted and TLR4 sensitivity is activated in microglia; FCGR2B is suppressed and the microglial uptake of pathogenic Aβ oligomers is then limited; VAV3 is repressed and KANK1 is activated, inhibiting RhoA activation and Rac1 signaling [61]. KATNAL1 is promoted, the microtubule-separating enzyme Katanin is activated, the protector TAU separated from the microtubules is redirected to the neurofibrillary tangles, leading to destabilization of the microtubules.

In AD neurons, mitochondria are destabilized. With respect to the presented model, PDZD8 and mitochondrial CAPN13 are promoted, Ca^2+^ is more effectively transported into mitochondria (PDZD8), and calpain cleaves mitochondrial proteins. Mitochondrial NADPH (MTHFD2L) is reduced and mitochondria are then not protected from ROS, conversely, ROS are supported by higher Cu^+^ concentrations (supported by SCO1); and KIF1B promotion increases mitochondrial density along the axon and overloads anterograde movement. As a result, axonal mitochondria as a source of “energy” and translation “support” are not functional, microtubules are destabilized, the transport of new mitochondria to the axon is blocked, and retrograde mitophagy (FYCO1, SMCR8, DENND2C) is downregulated.

The VCP chaperone is known to localize together with TAU, and changes in VCP can cause abnormal accumulation of TAU [85]. VCP is a multistage regulator of autophagy [116]; with respect to the presented model, the methylation of VCP to Lys315 is suppressed in AD (VCPKMT, Figure 6). However, the effect of methylation on VCP function and stability is currently unknown.

ELAVL/Hu genes/proteins and DCP2 are known to regulate RNA processing, transport, and stability. According to the presented model, DCP2 and ELAVL2/HuB are suppressed. DCP2 is a key component of an mRNA-decapping complex, and ELAVL2/HuB may be required for learning and memory [111]. Their repression (Figure 6) can significantly destabilize post-transcriptional “signal noise suppression”, leading to neuronal dysfunction and memory loss.

## Data Availability

Not applicable.

## Supplementary Materials

The sequence supporting information can be downloaded at: https://www.mdpi.com/article/10.3390/cimb44080243/s1, Figure S1: The most evolutionarily conserved sequence, from *S. cerevisiae* to Homo sapiens.
