# Supplementary material for: 1-L Transcription in Alzheimer’s Disease
Source: Curr Issues Mol Biol. 2022 Aug 9;44(8):3533–51. doi: 10.3390/cimb44080243 (PMC9406503; doi:10.3390/cimb44080243)
Supplement: Supplementary file 1 [file cimb-44-00243-s001.zip › cimb-1817881-supplementary.pdf]

Supplementary, *Current Issues in Molecular Biology*,

1-L Transcription in Alzheimer's Disease

Jozef Nahalka

[1] *Institute of Chemistry, Centre for Glycomics, Slovak Academy of Sciences, Dubravska Cesta 9, SK-84538 Bratislava, Slovakia;* [2] *Institute of Chemistry, Centre of Excellence for White-Green Biotechnology, Slovak Academy of Sciences, Trieda Andreja Hlinku 2, SK-94976 Nitra, Slovakia*

**Correspondence: nahalka@savba.sk*

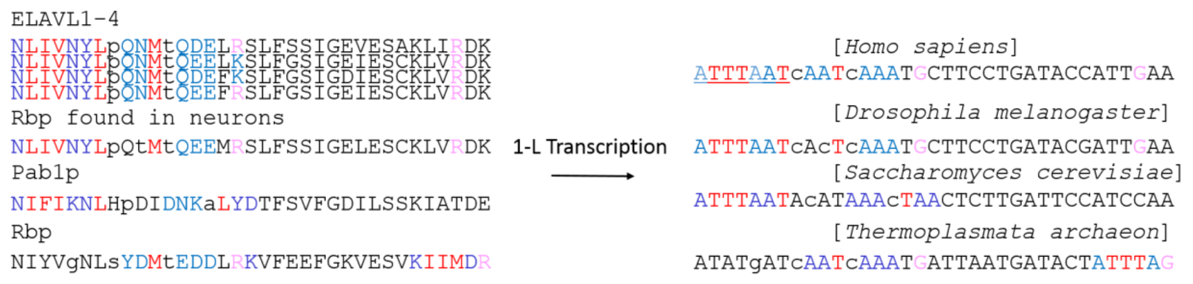

1 Supplementary Figure S1

2 Supplementary sequence information

```
>NP_958817.1 amyloid-beta precursor protein isoform c precursor [Homo
sapiens] APP
MLPGLALLLLAAWTARALEVPTDGNAGLLAEPQIAMFCGRLNMHMNVQNGKWDSDPSGTKTCIDTKEGILQYCQE
VYPELQITNVVEANQPVTIQNWCKRGRKQCKTHPHFVIPYRCLVGEFVSDALLVPDKCKFLHQERMDVCETHLHW
HTVAKETCSEKSTNLHDYGMLLPCGIDKFRGVEFVCCPLAEESDNVDSADAEEDDSDVWWGGADTDYADGSEDKV
VEVAEEEEVAEVEEEEADDDEDDEDGDEVEEEAEEPYEEATERTTSIATTTTTTTESVEEVVRVPTTAASTPDAV
DKYLETPGDENEHAHFQKAKERLEAKHRERMSQVMREWEEAERQAKNLPKADKKAVIQHFQEKVESLEQEAANER
QQLVETHMARVEAMLNDRRRLALENYITALQAVPPRPRHVFNMLKKYVRAEQKDRQHTLKHFEHVRMVDPKKAAQ
IRSQVMTHLRVIYERMNQSLSLLYNVPAVAEEIQDEVDELLQKEQNYSDDVLANMISEPRISYGNDALMPSLTET
KTTVELLPVNGEFSLDDLQPWHSFGADSVPANTENEVEPVDARPAADRGLTTRPGSGLTNIKTEEISEVKMDAEF
RHDSGYEVHHQKLVFFAEDVGSNKGAIIGLMVGGVVIATVIVITLVMLKKKQYTSIHHGVVEVDAAVTPEERHLS
KMQQNGYENPTYKFFEQMQN
```

> Amyloid beta 42 peptide, Aβ-42 peptide

```
DAEFRHDSGYEVHHQKLVFFAEDVGSNKGAIIGLMVGGVVIA
```

Aβ-42 / 1-L transcription S=C, S (Ser) transcription to C (cytidine)

```
ACATGAACGAATAAAATTTTCAATGCAAGCTTGTTTGGTTTC
```

```
>NM_001136180.2 Homo sapiens heat shock factor binding protein 1 like 1
(HSBP1L1), mRNA
Query  10   AATAAAATTTTCAATGCAAGCTTG  33
            |||| |||||||||||| ||||||
Sbjct  294  AATAGAATTTTCAATGCCAGCTTG  271
```

```
>XM_017000054.1 PREDICTED: Homo sapiens vav guanine nucleotide exchange
factor 3 (VAV3), transcript variant X2, mRNA
Query  11    ATAAAATTTTCAATGCAAGCTTGTTTGGTTTC  42
```

```
             ||||||||||||||| |   ||||||| ||||
Sbjct  3697  ATAAAATTTTCAATGAA---TTGTTTGCTTTC  3669

>NM_032119.4 Homo sapiens adhesion G protein-coupled receptor V1 (ADGRV1),
transcript variant 1, mRNA
Query  13    AAAATTTTCAATGCAA---GCTTGTTTGGTTTC  42
             |||| |  ||||||||   ||||||||||||||
Sbjct  4798  AAAAGTGACAATGCAAATGGCTTGTTTGGTTTC  4830

>NM_001100399.2 Homo sapiens PDS5 cohesin associated factor A (PDS5A),
transcript variant 1, mRNA
Query  9    GAATAAAATTTTCAATGCAAGCTT  32
            |||||||| ||||| ||||| |||
Sbjct  991  GAATAAAAATTTCATTGCAATCTT  968

>NM_014442.3 Homo sapiens sialic acid binding Ig like lectin 8 (SIGLEC8),
transcript variant 1, mRNA
Query  13    AAAATTTTCAATGCAAGCT  31
             ||||| |||||||||||||
Sbjct  2188  AAAATATTCAATGCAAGCT  2170

>NM_005725.6 Homo sapiens tetraspanin 2 (TSPAN2), transcript variant 1,
mRNA
Query  10    AATAAAATTTTCAATG  25
             ||||||||||||||||
Sbjct  1991  AATAAAATTTTCAATG  2006

>NM_015065.3 Homo sapiens exophilin 5 (EXPH5), transcript variant 1, mRNA
Query  14    AAATTTTCAATGCAAGCTTGTTTGGT  39
             || ||||||||||||  |||| ||||
Sbjct  8619  AACTTTTCAATGCAAATTTGTATGGT  8644

>NM_198459.4 Homo sapiens DENN domain containing 2C (DENND2C), transcript
variant 2, mRNA
Query  16    ATTTTCAATGCAAGCTTGTTTGGTTT  41
             ||||||||| ||||||||  || |||
Sbjct  1341  ATTTTCAATACAAGCTTGGGTGCTTT  1316

>NM_004589.4 Homo sapiens synthesis of cytochrome C oxidase 1 (SCO1), mRNA;
nuclear gene for mitochondrial product
Query  4     TGAACGAATAAAATTTTCAAT  24
             |||| ||| ||||||||||||
Sbjct  5272  TGAAGGAAGAAAATTTTCAAT  5292

>XM_017011793.2 PREDICTED: Homo sapiens polypeptide N-
acetylgalactosaminyltransferase like 5 (GALNTL5), transcript variant X1,
mRNA
Query  8     CGAATAAAATTTTCAATGCAA  28
             |||||||| ||||| ||||||
Sbjct  1160  CGAATAAAGTTTTCCATGCAA  1140
```

Reverse Aβ-42 / 1-L transcription S=C, S (Ser) transcription to C (cytidine)

CTTTGGTTTGTTCGAACGTAACTTTTAAAATAAGCAAGTACA

```
>XM_017018372.2 PREDICTED: Homo sapiens SET binding factor 2 (SBF2),
transcript variant X4, mRNA
Query  3     TTGGTTTGTTC-GAACGTAACTTTTAAAAT  31
             ||| ||||||  |||| |||||||||||||
```

```
Sbjct  1119  TTGCTTTGTTGTGAACATAACTTTTAAAAT  1090

>NM_003884.5 Homo sapiens lysine acetyltransferase 2B (KAT2B), mRNA
Query  20    AACTTTTAAAATAAGCA  36
             |||||||||||||||||
Sbjct  3153  AACTTTTAAAATAAGCA  3169

>NM_133334.2 Homo sapiens nuclear receptor binding SET domain protein 2
(NSD2), transcript variant 7, mRNA
Query  22    CTTTTAAAATAAGCAAG  38
             |||||||||||||||||
Sbjct  6161  CTTTTAAAATAAGCAAG  6145

>XR_945930.3 PREDICTED: Homo sapiens family with sequence similarity 245
member B (FAM245B), transcript variant X1, ncRNA
Query  22   CTTTTAAAATAAGCAAG  38
            |||||||||||||||||
Sbjct  242  CTTTTAAAATAAGCAAG  258

>NM_004001.5 Homo sapiens Fc gamma receptor IIb (FCGR2B), transcript
variant 1, mRNA
Query  8     TTGTTCGAACGTAACTTTTAAAATAAGCA  36
             ||||||  ||||||| | ||| |||||||
Sbjct  1363  TTGTTCTCACGTAACCTGTAACATAAGCA  1335

>NM_203330.2 Homo sapiens CD59 molecule (CD59 blood group) (CD59),
transcript variant 1, mRNA
Query  18    GTAACTTTTAAAATAAGCA  36
             ||||| |||||||||||||
Sbjct  1475  GTAACATTTAAAATAAGCA  1457

>NM_004432.5 Homo sapiens ELAV like RNA binding protein 2 (ELAVL2),
transcript variant 1, mRNA
Query  14    GAACGTAACTTTTAAAATA  32
             |||||||||||| ||||||
Sbjct  2754  GAACGTAACTTTCAAAATA  2736

>NM_152624.6 Homo sapiens decapping mRNA 2 (DCP2), transcript variant 1,
mRNA
Query  10    GTTCGAACGTAACTTTTAAAA  30
             ||| ||||||||| |||||||
Sbjct  3960  GTTGGAACGTAACATTTAAAA  3940

>NM_173791.5 Homo sapiens PDZ domain containing 8 (PDZD8), mRNA
Query  27    AAAATAAGCAAGTACA  42
             ||||||||||||||||
Sbjct  3556  AAAATAAGCAAGTACA  3571

>XM_017020407.2 PREDICTED: Homo sapiens methyltransferase 21C, AARS1 lysine
(METTL21C), transcript variant X1, mRNA
Query  22    CTTTTAAAATAAGCAA  37
             ||||||||||||||||
Sbjct  3535  CTTTTAAAATAAGCAA  3550
```

Aβ-42 / 1-L transcription S=G, S (Ser) transcription to G (guanosine)

ACATGAAGGAATAAAATTTTCAATGGAAGCTTGTTTGGTTTC

```
>NM_152703.5 Homo sapiens sterile alpha motif domain containing 9 like
(SAMD9L), transcript variant 1, mRNA
Query  2     CATGAAGGAATAAAA-TTTTCA  22
             ||||||||||||||| ||||||
Sbjct  3904  CATGAAGGAATAAAAGTTTTCA  3883

>NM_001005190.2 Homo sapiens olfactory receptor family 7 subfamily A member
10 (OR7A10), mRNA
Query  3     ATGAAGGAATAAAATTTTCAAT  24
             |||| |||||| ||||||||||
Sbjct  1559  ATGACGGAATATAATTTTCAAT  1580

>NM_017672.6 Homo sapiens transient receptor potential cation channel
subfamily M member 7 (TRPM7), transcript variant 1, mRNA
Query  6     AAGGAATAAAATTTTC-----AATGGAAGCTTGTTTGGTTTC  42
             |||| |||||| ||||     | ||||||||| |||| ||||
Sbjct  2807  AAGGCATAAAACTTTCGCGTAATTGGAAGCTTTTTGATTTC  2766

>NM_014616.3 Homo sapiens ATPase phospholipid transporting 11B (putative)
(ATP11B), mRNA
Query  2     CATGAAGGAATAAAATTTTCAATGGAA  28
             ||| ||||||| | |||||||| ||||
Sbjct  5310  CATAAAGGAATTACATTTTCAAGGGAA  5284

>XM_011517508.2 PREDICTED: Homo sapiens lymphocyte antigen 96 (LY96),
transcript variant X3, mRNA
Query  8    GGAATAAAATTTTCAATGGAAG  29
            |||||||||||||| | |||||
Sbjct  414  GGAATAAAATTTTCTAAGGAAG  435

>XM_017004664.1 PREDICTED: Homo sapiens sodium voltage-gated channel alpha
subunit 3 (SCN3A), transcript variant X7, mRNA
Query  7     AGGAATAAAATTTTCAATGGAA  28
             || ||||||||| |||||||||
Sbjct  5191  AGAAATAAAATTATCAATGGAA  5212

>NM_080661.6 Homo sapiens glycine-N-acyltransferase like 1 (GLYATL1),
transcript variant 1, mRNA
Query  10    AATAAAATTTTCAATGGAA  28
             ||||||||||||||| |||
Sbjct  1409  AATAAAATTTTCAATAGAA  1427

>NM_003617.4 Homo sapiens regulator of G protein signaling 5 (RGS5),
transcript variant 1, mRNA
Query  4     TGAAGGAATAAAATTTTCAATGGAAGCTT  32
             || ||||||| |||||| |||| ||| ||
Sbjct  3035  TGTAGGAATACAATTTTAAATGTAAGATT  3007

>NM_001004346.4 Homo sapiens methylenetetrahydrofolate dehydrogenase (NADP+
dependent) 2 like (MTHFD2L), transcript variant 1, mRNA
Query  12    TAAAATTTTCAATGGAAGCTTG-TTTGGTT  40
             ||||||||||| |||||  ||| |||||||
Sbjct  1321  TAAAATTTTCATTGGAA--TTGTTTGGTT  1294

>NM_025027.4 Homo sapiens zinc finger protein 606 (ZNF606), transcript
variant 1, mRNA
Query  1     ACATGAAGGAATAAAA  16
             ||||||||||||||||
```

```
Sbjct  3781  ACATGAAGGAATAAAA  3796

>NM_004125.4 Homo sapiens DNAJC25-GNG10 readthrough (DNAJC25-GNG10), mRNA
Query  4    TGAAGGAATAAAATTTTCAAT  24
            |||||||||| |||||| |||
Sbjct  717  TGAAGGAATAGAATTTTAAAT  737

>NM_001017998.4 Homo sapiens G protein subunit gamma 10 (GNG10), transcript
variant 1, mRNA
Query  4    TGAAGGAATAAAATTTTCAAT  24
            |||||||||| |||||| |||
Sbjct  421  TGAAGGAATAGAATTTTAAAT  441

>NM_182641.4 Homo sapiens bromodomain PHD finger transcription factor
(BPTF), transcript variant 1, mRNA
Query  16    ATTTTCAATGGAAGCTTGTTT  36
             |||||||||||||  ||||||
Sbjct  3783  ATTTTCAATGGAATTTTGTTT  3763

>NM_021255.3 Homo sapiens pellino E3 ubiquitin protein ligase family member
2 (PELI2), mRNA
Query  4     TGAAGGAATAAAATTTTCAAT  24
             ||||| |||||| ||||||||
Sbjct  5575  TGAAGAAATAAACTTTTCAAT  5555

>NM_144775.3 Homo sapiens SMCR8-C9orf72 complex subunit (SMCR8), mRNA
Query  8     GGAATAAAATTTTCAA  23
             ||||||||||||||||
Sbjct  4682  GGAATAAAATTTTCAA  4667

>XM_017022414.2 PREDICTED: Homo sapiens mitogen-activated protein kinase
kinase 5 (MAP2K5), transcript variant X5, mRNA
Query  5     GAAGGAATAAAATTTTCAA--TGGAAGCTT  32
             |||||  ||||||||||||  | |||||||
Sbjct  1949  GAAGGGTTAAAATTTTCAACTTAGAAGCTT  1920

>NR_037883.1 Homo sapiens POU3F3 adjacent non-coding transcript 1 (PANTR1),
transcript variant 1, long non-coding RNA
Query  13   AAAATTTTCAATGGAAGCTTGTTTGG  38
            ||| |||| |||||||||||  ||||
Sbjct  291  AAACTTTTAAATGGAAGCTTTATTGG  316
```

Reverse Aβ-42 / 1-L transcription S=G, S (Ser) transcription to G (guanosine)

CTTTGGTTTGTTCGAAGGTAACTTTTAAAATAAGGAAGTACA

```
>NM_015176.4 Homo sapiens F-box protein 28 (FBXO28), transcript variant 1,
mRNA
Query  4     TGGTTTGTTCG-AAGGTAACTTTTAAAATAA  33
             ||||||||| | ||||||| |||||||||||
Sbjct  2878  TGGTTTGTTGGGAAGGTAA-TTTTAAAATAA  2907

>NM_207015.3 Homo sapiens N-acetylated alpha-linked acidic dipeptidase like
2 (NAALADL2), mRNA
Query  19    TAACTTTTAAAATAAGGAAGTACA  42
             ||||||||||||||||| ||| ||
Sbjct  8383  TAACTTTTAAAATAAGGTAGTCCA  8360
```

```
>NM_024852.4 Homo sapiens argonaute RISC catalytic component 3 (AGO3),
transcript variant 1, mRNA
Query  19     TAACTTTTAAAATAAGGAAGTACA  42
              |||||||||||||||  |||||||
Sbjct  11234  TAACTTTTAAAATAA--AAGTACA  11255

>NM_024513.4 Homo sapiens FYVE and coiled-coil domain autophagy adaptor 1
(FYCO1), transcript variant 1, mRNA
Query  21    ACTTTTAAAATAAGGAAGTACA  42
             |||||||||||||  |||||||
Sbjct  8113  ACTTTTAAAATAATAAAGTACA  8134

>NM_015158.5 Homo sapiens KN motif and ankyrin repeat domains 1 (KANK1),
transcript variant 1, mRNA
Query  23    TTTTAAAATAAGGAAGT  39
             |||||||||||||||||
Sbjct  4644  TTTTAAAATAAGGAAGT  4660

>NM_032116.5 Homo sapiens katanin catalytic subunit A1 like 1 (KATNAL1),
transcript variant 1, mRNA
Query  19    TAACTTTTAAAATAAGGAAGTA  40
             ||||||||||||||| ||| ||
Sbjct  3411  TAACTTTTAAAATAAAGAAATA  3432

>NM_153836.4 Homo sapiens cellular repressor of E1A stimulated genes 2
(CREG2), mRNA
Query  18    GTAACTTTTAAAATAAG  34
             |||||||||||||||||
Sbjct  3543  GTAACTTTTAAAATAAG  3527

>NM_012072.4 Homo sapiens CD93 molecule (CD93), mRNA
Query  15    AAGGTAACTTTTAAAATAAGGA  36
             || |||||||||||||| ||||
Sbjct  3405  AATGTAACTTTTAAAATGAGGA  3384

>XM_011533161.3 PREDICTED: Homo sapiens calpain 13 (CAPN13), transcript
variant X6, mRNA
Query  18    GTAACTTTTAAAATAAGGAAGT  39
             ||||||||| |||| |||||||
Sbjct  2649  GTAACTTTTGAAATTAGGAAGT  2670

>NM_001362797.2 Homo sapiens zinc fingers and homeoboxes 2 (ZHX2),
transcript variant 1, mRNA
Query  19    TAACTTTTAAAATAAGGAA  37
             |||||||||||| ||||||
Sbjct  4309  TAACTTTTAAAAGAAGGAA  4327

>XM_017021640.2 PREDICTED: Homo sapiens valosin containing protein lysine
methyltransferase (VCPKMT), transcript variant X2, mRNA
Query  15    AAGGTAACTTTTAAAATAA  33
             |||||||||||||||| ||
Sbjct  5051  AAGGTAACTTTTAAAAAAA  5033

>NM_024524.4 Homo sapiens ATPase 13A3 (ATP13A3), transcript variant 1, mRNA
Query  15    AAGGTAACTTTTAAAA  30
             ||||||||||||||||
Sbjct  5510  AAGGTAACTTTTAAAA  5525
```

```
>NM_015074.3 Homo sapiens kinesin family member 1B (KIF1B), transcript
variant 1, mRNA
Query  11    TTCGAAGGTAACTTTTAAAAT  31
             || |||||| |||||||||||
Sbjct  8077  TTTGAAGGTCACTTTTAAAAT  8097

>XM_017008195.1 PREDICTED: Homo sapiens coiled-coil serine rich protein 1
(CCSER1), transcript variant X9, mRNA
Query  20    AACTTTTAAAATAAGGAAGTA  40
             || |||||||||| |||||||
Sbjct  6426  AAATTTTAAAATATGGAAGTA  6446
```
